# 

**Published:** 1872-05

**Authors:** 


ADVERTISING SHEET.—A large advertising sheet will be commenced with the next number of the JOURNAL.

